# Supplementary material for: Associations between women’s empowerment and children’s health status in Ethiopia
Source: PLoS One. 2020 Jul 20;15(7):e0235825. doi: 10.1371/journal.pone.0235825 (PMC7371184; doi:10.1371/journal.pone.0235825)
Supplement: S1 Fig — (DOCX) [file pone.0235825.s001.docx]

S1 Fig. Scree plot of eigenvalues after factor analysis
