# Supplementary figures and images for: Associations between women’s empowerment and children’s health status in Ethiopia
Source: PLoS One. 2020 Jul 20;15(7):e0235825. doi: 10.1371/journal.pone.0235825 (PMC7371184; doi:10.1371/journal.pone.0235825)

S2 Fig. Confirmatory factor analysis model for women’s empowerment (EDHS-2016).


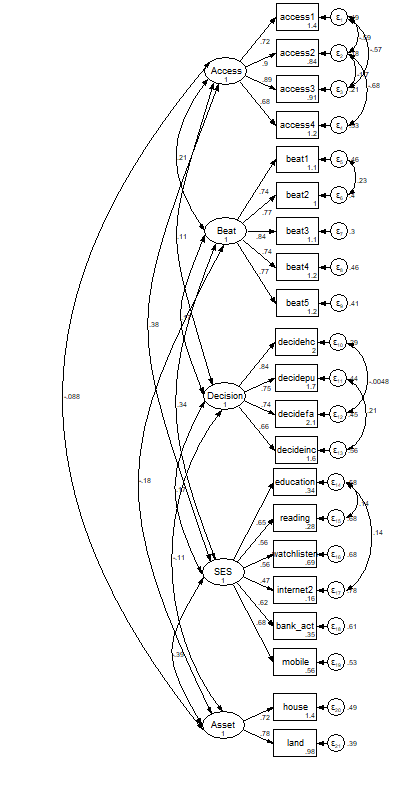

Supplement: S2 Fig — (DOCX) [file pone.0235825.s002.docx]
