# Supplementary material for: Associations between women’s empowerment and children’s health status in Ethiopia
Source: PLoS One. 2020 Jul 20;15(7):e0235825. doi: 10.1371/journal.pone.0235825 (PMC7371184; doi:10.1371/journal.pone.0235825)
Supplement: S1 Table — (DOCX) [file pone.0235825.s003.docx]

S1 Table. Result of EFA (Rotated factor loadings)

| **Indicators** | **Beat** | **SES** | **Access** | **Decision** | **Asset** | **Uniqueness** |
| --- | --- | --- | --- | --- | --- | --- |
| Health care decisions | -0.0025 | -0.0167 | 0.0203 | **0.8566** | -0.0160 | 0.2647 |
| Large household purchases | -0.0188 | 0.0110 | 0.0028 | **0.8540** | 0.0227 | 0.2752 |
| Visits to family or relatives | 0.0339 | -0.0208 | -0.0034 | **0.7991** | -0.0150 | 0.3546 |
| Money husband earns | -0.0140 | 0.0320 | -0.0259 | **0.7973** | 0.0041 | 0.3637 |
| Beating justified when wife goes out without telling a husband | **0.8247** | 0.0114 | 0.0279 | -0.0211 | 0.0518 | 0.3187 |
| Beating justified when wife neglects the children | **0.8570** | -0.0181 | 0.0009 | -0.0121 | 0.0122 | 0.2786 |
| Beating justified when wife argues with husband | **0.8630** | -0.0000 | -0.0265 | -0.0117 | -0.0310 | 0.2592 |
| Beating justified when wife refuses to have sex with husband | **0.7783** | 0.0153 | -0.0027 | 0.0237 | -0.0175 | 0.3781 |
| Beating justified when wife burns the food | **0.8084** | 0.0048 | 0.0029 | 0.0232 | -0.0130 | 0.3338 |
| Getting permission to go | -0.0024 | -0.0717 | **0.8060** | 0.0250 | 0.0807 | 0.3810 |
| Getting money needed for treatment | 0.0053 | 0.0860 | **0.7061** | 0.0292 | 0.0433 | 0.4549 |
| Distance to a health facility | -0.0106 | 0.0478 | **0.7920** | 0.0010 | -0.0577 | 0.3374 |
| Not wanting to go alone | 0.0086 | -0.0655 | **0.8300** | -0.0434 | -0.0217 | 0.3389 |
| Highest educational level | 0.0122 | **0.7771** | -0.0438 | 0.0217 | -0.0095 | 0.4014 |
| Frequency of reading newspaper | -0.0214 | **0.7412** | -0.0492 | -0.0061 | 0.0473 | 0.4962 |
| watching television and listening to a radio | -0.0235 | **0.5279** | 0.1377 | -0.0109 | -0.1140 | 0.6171 |
| Frequency of using internet last month | -0.0026 | **0.6902** | -0.0925 | -0.0082 | 0.0938 | 0.5830 |
| Has an account in a bank | 0.0033 | **0.7016** | 0.0194 | -0.0041 | 0.0303 | 0.5095 |
| Owns a mobile telephone | 0.0420 | **0.6234** | 0.0987 | -0.0027 | -0.1032 | 0.4965 |
| Owns a house alone or jointly | 0.0018 | 0.0310 | 0.0126 | -0.0155 | **0.8881** | 0.2254 |
| Owns land alone or jointly | 0.0004 | -0.0150 | 0.0130 | 0.0133 | **0.8700** | 0.2395 |
| **Cronbach’s Alpha** | **0.88** | **0.74** | **0.79** | **0.84** | **0.71** | **0.8214** |
