# Supplementary material for: Associations between women’s empowerment and children’s health status in Ethiopia
Source: PLoS One. 2020 Jul 20;15(7):e0235825. doi: 10.1371/journal.pone.0235825 (PMC7371184; doi:10.1371/journal.pone.0235825)
Supplement: S2 Table — (DOCX) [file pone.0235825.s004.docx]

S2 Table. The difference in distribution of the five dimensions of women’s empowerment by the four child health outcomes.

| **Variable** | **Stunted** | | | **Wasted** | | | **Pneumonia** | | | **Anemia** | | |
| --- | --- | --- | --- | --- | --- | --- | --- | --- | --- | --- | --- | --- |
| Women empowerment greater than the median | No  (Freq.) | Yes  (Freq.) | Pr. | No  (Freq.) | Yes  (Freq.) | Pr. | No  (Freq.) | Yes  (Freq.) | Pr. | No  (Freq.) | Yes  (Freq.) | Pr. |
| **Access** |  |  |  |  |  |  |  |  |  |  |  |  |
| No | 2,716 | 1,716 | 0.000 | 3,866 | 594 | 0.001 | 4,464 | 496 | 0.000 | 1,455 | 2,445 | 0.000 |
| Yes | 2,916 | 1,507 | 0.000 | 3,973 | 486 | 0.001 | 4,564 | 395 | 0.000 | 1,649 | 2,246 | 0.000 |
| **Beat** |  |  |  |  |  |  |  |  |  |  |  |  |
| No | 2,668 | 1,760 | 0.000 | 3,868 | 594 | 0.001 | 4,464 | 496 | 0.000 | 1,534 | 2,364 | **0.413** |
| Yes | 2,964 | 1,463 | 0.000 | 3,971 | 486 | 0.001 | 4,564 | 395 | 0.000 | 1,570 | 2,327 | **0.413** |
| **Decision** |  |  |  |  |  |  |  |  |  |  |  |  |
| No | 2,738 | 1,696 | 0.000 | 3,885 | 578 | 0.016 | 4,590 | 499 | 0.004 | 1,476 | 2,422 | 0.000 |
| Yes | 2,894 | 1,527 | 0.000 | 3,954 | 502 | 0.016 | 4,438 | 392 | 0.004 | 1,628 | 2,269 | 0.000 |
| **SES** |  |  |  |  |  |  |  |  |  |  |  |  |
| No | 2,578 | 1,851 | 0.000 | 3,856 | 610 | 0.000 | 4,642 | 460 | **0.933** | 1,481 | 2,417 | 0.001 |
| Yes | 3,054 | 1,372 | 0.000 | 3,983 | 470 | 0.000 | 4,386 | 431 | **0.933** | 1,623 | 2,274 | 0.001 |
| **Asset** |  |  |  |  |  |  |  |  |  |  |  |  |
| No | 2,984 | 1,445 | 0.000 | 3,926 | 534 | **0.718** | 4,514 | 446 | **0.997** | 1,505 | 2,404 | 0.018 |
| Yes | 2,648 | 1,778 | 0.000 | 3,913 | 546 | **0.718** | 4,514 | 445 | **0.997** | 1,599 | 2,287 | 0.018 |

Pr. Represents p-value for Pearson chi2
