# Supplementary material for: Associations between women’s empowerment and children’s health status in Ethiopia
Source: PLoS One. 2020 Jul 20;15(7):e0235825. doi: 10.1371/journal.pone.0235825 (PMC7371184; doi:10.1371/journal.pone.0235825)
Supplement: Attachment — Submitted filename: Response to Reviewers.docx [file pone.0235825.s005.docx]

# **Associations between Women’s Empowerment and Children’s Health Status in Ethiopia**

**Response to the reviews**

Dear Editor,

We hereby resubmit our manuscript entitled “*Associations between Women’s Empowerment and Child Health Status in Ethiopia”* by incorporating revisions based on the comments and suggestions of the two reviewers. We would like to thank the editor and the reviewers for careful and thorough reading of this manuscript and for the constructive comments and suggestions, which help to improve the quality of the manuscript. We believe that the changes have considerably improved the manuscript argumentation and clarity and appreciate the efforts of the reviewers to this extent. Below we describe in detail the changes we have made and respond to the reviewer's comments. For easy reference, we create a table to present a reviewer’s comment and our response.

# **Reviewer # 1**

| **No** | **Reviewer’s Comment** | **Author’s response** |
| --- | --- | --- |
| 1 | I was very pleased to be invited to review this paper. The authors demonstrate a fairly good understanding of the literature regarding women’s empowerment and child outcomes, though they may be overstating that it is limited, as many of the existing articles are not cited. They could possible specify that it is limited in this particular context. They have also approached their impressive analytic intentions with the appropriate level of data, namely DHS. The extensive and thorough measures of female empowerment is the contribution of the paper, but the introduction, literature review, and conclusion seriously limit the implications of this paper. There needs to be a deeper dive into the existing literature, the findings of this paper, and the implications for a variety of stakeholders. | Thank you for your interest to review our paper and for giving us valuable comments and suggestions to improve our paper considerably. The paper has now addressed all these concerns by addressing the specific comments provided below. |
| 2 | **Section 1:** The second paragraph about rates could be more succinctly presented. The authors do a good job of explaining the gap that exists, they just need to add more of the existing literature to the review in order to convincingly say that this gap exists across a majority of papers on this topic. | We have now added one paragraph presenting literature on the prevalence of wasting, stunting, anemia and pneumonia (see paragraph three of the introduction section) . |
| 3 | **Section 2:** The authors are thorough in their discussion of Ethiopia. While context is important, this should be presented more succinctly and replaced with information about the existing studies on women’s empowerment and child health. If this paper is indeed addressing the gap in this pretty expansive literature; I need to be convinced that this gap exists. Section 3 hints at this in the first paragraph, but it does not adequately situation this paper in the existing literature. | Thanks for pointing out this comment. We have taken this comment into consideration and have made another review of existing studies on women’s empowerment and child health in Ethiopia and we added one paragraph in the revised version of the manuscript (see paragraph 4 of section 2). The newly added studies are: Jones et al. (2019), Heckert et al. (2019), Kuche et al. (2018), Na et al 2015 and Yimer and Tadesse (2015). |
| 4 | **Section 4.1.2:** It is helpful to present alpha scores.  What is the justification for the all or nothing coding of the decision-making variable?  It is not clear if the barriers to healthcare are combined into one variable.  There are two paragraphs that explain the attitudes toward wife-beating  It would be helpful to justify (citations) why exposure to media is a measure of socio-economic empowerment. | Cronbach’s alpha scores were reported in the Method section in the previous version of the manuscript (See Section 4.2.1). Our model had Cronbach’s alpha coefficient ranges from 0.78 to 0.88 suggesting good reliability or internal consistency.  Coding of women’s decision-making variables determined based on their involvement in the household decision making power. We considered women who make decision alone or jointly with their partner as empower, otherwise the women considered to be disempowered. We did this following previous studies (see e.g., (Mabsout, 2011, Ndaimani et al (2018), Hearld et al (2018), Wado (2018). This has now included on the previous version of the manuscript (see paragraph 1 of section 4.1.2).  Yes, the barriers to access healthcare were combined into one single latent variable. We have now revised this part of the text to make it clearer to the reader.  We have now removed the repeated paragraph about wife-beating in the revised version of the manuscript.  Women’s exposure to media represents women economic status of women in Ethiopia, and following Wado (2018), we included them in the domain of women’s socio-economic variables. We have now added a reference as a justification for the grouping. |
| 5 | **Section 5.2:** Numbers should be used in the results opposed to just positively or negatively associated. | We have presented the number for the associations mentioned in the text throughout the manuscript. |
| 6 | **Section 6.1:** There was no mention of a bank account or mobile phone in the measures section. | Women’s ownership of a bank account and mobile phone is included under the socio-economic domain of women’s empowerment. This was presented in the appendix S 1 Table 1 of the previous version of the manuscript, but were not defined in the text.. Now we explain the two indicators of gender empowerment in the text (see section 4.1.2 in the revised version of the manuscript). Full list of women’s empowerment indicators used in this study with their descriptions and summary statistics are presented appendix S 1 Table 1. |
| 7 | **Section 7:** A main contribution of this paper is the thorough investigation of different avenues of women’s empowerment. However, the literature review and the conclusion does not convince me that they are covering all important measures of empowerment or how those different measures would/do work differently to influence child health. Why could certain empowerment measures matter over others for child’s health? The conclusion is seriously lacking considering the numerous findings, which are the actually, most measures of empowerment don’t statistically seem to matter. This could be a troubling take away, so explaining it is of utter importance. | Now we have added relevant and recent new studies regarding women’s empowerment and child health status in section 1, 2 and the discussion section. Studies includes: Jones et al. (2019), Heckert et al. (2019), Kuche et al. (2018), Na et al 2015 and Yimer and Tadesse (2015), Alemayehu et al (2015), Fantahun et al, (2007). |
| 8 | Typographical and grammar edits- this paper needs a thorough read through, particularly the discussion and conclusion sections. A few are mentioned below, but there is a need for greater clarity at the sentence level.  pg 2 line 28 “Even those that with an…”  pg 3 line 59 “Reducing THE child mortality rate’  pg 3 line 70 “helps improving”  pg 7 line 143 “with a greater agency”  Pg 25 lines 522-525 do not use consistent citations  Pg 26 lines 552-556 are not clear  Pg 28 line 583 “giving the…” | The indicated grammatical issues have now been corrected. On top of this, the revised manuscript has been checked by a professional English editor. |

# **Reviewer # 2**

| **No** | **Reviewer’s Comment** | **Author’s response** |
| --- | --- | --- |
| 1 | **Overall:** The paper uses the latest Ethiopian DHS to examine the association between women’s empowerment and child health status. The authors use SEM to fit a measurement and a structural component and to simultaneously examine how women’s empowerment is associated with both anthropometric and non-anthropometric child health outcomes. The findings contribute to the evidence base on how women’s empowerment is associated with different child health outcomes and can help inform local interventions to help improve women’s empowerment and child health. | Thank you for your interest to review our paper and for giving us valuable comments and suggestions to improve our paper considerably. |
| 2 | **Major concerns**  Measurement model – it is unclear why the authors conceptualize and fit two separate anthropometric and non-anthropometric latent health variables. It is also not clear why these constructs are conceptualized as latent construct rather than as observed variables which is more common in the literature on women’s empowerment and children’s health status. Additionally, why was weight-for-age Z-score not included in the anthropometric latent construct? | We separate the two latent health outcomes for two reasons. First, All the indicators considered in our study are not very similar to group them in one category. So, we identified two separate groups, i.e., one represents the children’s anthropometric indicators (height-for-age z-score and weight-for-height z-score) and the other is from other outcomes such as infectious and communicable diseases  (Pneumonia and anemia). Second, we are interested to compare the association of women’s empowerment on these separate latent health outcomes (See section 3 paragraph 4).  Since we have two indicators for each of the health categories and gender empowerment dimensions, we had to create a composite index for each of the categories, we construct a latent variable for both for health outcomes and gender empowerment dimensions following previous studies  Desai and Johnson (2005), Mabsout (2011), Zereyesus et al (2017), Ndaimani (2018).  We exclude weight-for-age z-score from the latent construct because it failed to converge during estimation of the MIMIC model due to highly correlated with both weight-for-height and height-for-age. This is because weight-for-age Z-score is a composite index of weight-for-height and height-for-age meaning that weight-for-age Z-score provides redundant information in the constructed latent index. So, we exclude this indicator from our analysis. |
| 3 | Several details are not included in the methods section, which would make replication of the results difficult. Was half the sample used for EFA? If so, how was this sample selected? How was the cut-off of 0.3 for keeping factor loadings chosen?  It would be helpful to include all factor loadings in supplemental table 3, and then indicate (in bold, italic or some symbol) which factor loadings made the cut-off. It is informative to know how close to the cut-off factor loadings were.  With respect to CFA, supplemental figure 2 shows that indicator error terms were allowed to be correlated. It is not clear from the methods section why this is the case; if this was an a priori hypothesis or if the authors tested multiple CFA models allowing/not allowing error terms to be correlated. What estimator was used to fit the CFA model? Further, the authors provide no evidence of discriminant validity, e.g. what are the correlations between factors. | We use the full sample to perform both EFA and CFA. To do this, first we performed EFA analysis by identifying and selecting all indicators related to women’s empowerment to identify and determine whether a set of indicators stand together on one latent dimension and to establish a set of latent women empowerment factors. After identifying women’s empowerment dimensions using EFA, CFA was applied to the same dataset using the identified five factors and 21 indicators from EFA. We used CFA verify the factor structure of the indicators related to women’s empowerment and to test the hypothesis that a relationship between observed indicators and their underlying latent constructs exists (see section 4.2.1 ).  We have modified and replaced Table 3 to include all factor loadings from factor analysis. Indicators with loadings >0.3 are indicated with bold (see S3 Table 2.).  We allowed some of the indicator error terms to be correlated to better fit the CFA model. To do this we have calculated the modification indices of the CFA model. Factors which have higher modification indices were allowed their error terms to have correlated each other. In this way we find the best fit CFA model. This has now explained in the method section. |
| 4 | Please indicate what software was used for data analysis. | Data analysis was performed using STATA version 14. We include this information in the manuscript. |
| 5 | How was missing data handled in the EFA, CFA and MIMICs models? | We perform both CFA and MIMIC models with missing values. |
| 6 | Line 407 specify in the methods section what test was used to test for differences in empowerment indices and how statistical significance was defined. It may be helpful to create a table with all the results rather than to selectively present some of them in text. | After describing the relationship between the five dimensions of women’s empowerment and child health outcomes using box plot, we conducted nonparametric equality-of-medians test to check the null hypothesis that the median empowerment indices between the category of child health outcomes are identical. Rejecting our null hypothesis means, the median of women’s empowerment is statistically significant and different. We have now presented a table showing the results of the nonparametric equality-of-medians test in the supplementary information S5 Table 3 of the revised version of the manuscript. |
| 7 | Table 2, the methods section should describe how the marginal effects of women’s empowerment on child’s health were calculated. The table is somewhat confusing with empowerment dimensions as rows but model 2 including the empowerment index. The results presented after Table 3 indicate that model 2 was the best fit model, but marginal effects are not calculated for this model. How come? | Comment taken. We added a description on how the marginal effect of women’s empowerment on child health is calculated. We have modified the table based on your comment and the marginal effect of model 2 for both anthropometric and non-anthropometric health indicator are included in the Table 3. As we discussed in result section, women’s empowerment with regard to socio-economic dimensions for anthropometric indicator and decision making for non-anthropometric indicator were found to be statistically significant. So, we calculate the marginal effect for the indicators related to such outcomes. We also included the overall empowerment index as rows with empowerment dimensions. |
| 8 | The authors should consider moving the measurement results and Table 3 before the MIMICs results, since the measurement results inform the outcomes which are then used in the MIMICs model. The health indicators listed in Table 3 are not in line with the definitions in the text. The authors should be more careful and consistent in how they refer to the health indicators throughout to improve the clarity of the article. | We have now moved “Table 3” before the MIMIC results and changed to “Table 1”. We also modified the name of the health indicators listed in this table according to the prior definitions in the paper. |
| 9 | The use of causal language is misleading. Given the cross-sectional nature of the data and the analytic methods employed, the authors can only assess the association between women’s empowerment and child health status. Throughout the manuscript, the authors should clean up the causal language (e.g., “impact”, “affect”, “effect”) and replace it with the appropriate non-casual language. | Point taken. Correction has been made throughout the manuscript accordingly. |
| 10 | The discussion on child, women and household characteristics associated with anthropometric and non-anthropometric health indicators can be cut down. These are largely known from the literature. The discussion will benefit from a more in-depth discussion on why certain domains of women’s empowerment are associated with child outcomes while others are not, and how these findings are, or are not, specific to Ethiopia. Lines 542-545 are a bit unclear with respect to the hypotheses the authors are making. The authors argue that “if the woman decides on their partner’s earnings, they may allocate their money for their children”. Yet the indicator for decision-making with respect to partner’s earnings was defined as 1=“respondent alone or jointly”, which does not necessarily substantiate the argument made by the authors. It might not be women’s ability to decide on earnings, but rather their involvement in the decision-making process. The discussion should be a bit more nuanced with respect to the interpretations and implications given the data limitations acknowledged by the authors. | Now we have added relevant and recent new studies regarding women’s empowerment and child health status in section 1, 2 and the discussion section. Studies includes: Jones et al. (2019), Heckert et al. (2019), Kuche et al. (2018), Na et al 2015 and Yimer and Tadesse (2015), Alemayehu et al (2015), Fantahun et al, (2007).  The reviewer is right in noting that the specification of our decision-making variable does not allow as it is defined as a dummy variable that 1=“women makes decision alone or jointly”. So, the specified text has been revised as “if the woman involves in making decisions on their partner’s earnings , they may allocate their money for their children”. |
| 11 | ***Minor concerns***  Overall – the manuscript can benefit from copyediting; there are typos, unclear sentences, etc. Further, the authors should try to use more consistent language to improve the clarity of the paper. For example, switching between nutrition indicators and anthropometric indicators make it unclear whether these are two distinct types of indicators. Please spell out all abbreviations the first time they are used, e.g. ARI line 263. | We have now avoided the term nutrition and replaced by anthropometric indicators throughout the manuscript. And we spell out all the abbreviation the first time we use them.  The manuscript has been checked by native English speaker. |
| 12 | Abstract – unclear whether “empowered women” refers to a binary variable where women were considered empowered or not empowered, or a continuous variable where higher empowerment was associated with lower likelihood of stunting. | Since our latent empowerment measures are factors where higher factors values indicate higher level of empowerment, we use the term higher empowerment. We revised this term the current version of the manuscript so that it becomes clearer to the reader. |
| 13 | Introduction – the introduction should be strengthened to provide relevant statistics on the prevalence of wasting, stunting, anemia and pneumonia as well as on the state of women’s empowerment. The local context is heavily described in terms of infant and child mortality, which are not outcomes the paper focuses on. Further, it would be helpful to provide more context on what previous studies, if any. in Ethiopia (neighboring countries or Sub-Saharan Africa) have found on the association between women’s empowerment and child health outcomes, and what are the specific gaps in the literature that the paper is trying to address. | We have now reviewed existing literature and added the most relevant papers related to our study. In the previous version of the manuscript, we discussed about infant and child mortality is to show the problems related to child in developing countries in general and in Ethiopia in particular. We have now extended this and added one paragraph explaining literatures regarding the prevalence of wasting, stunting, anemia and pneumonia. Kindly see paragraph three of the introduction part. We added previous studies investigating the associations between women’s empowerment and child health both in section 2 and in the discussion part of our manuscripts. For more see the new refences (Jones et al. (2019), Heckert et al. (2019), Kuche et al. (2018), Na et al 2015 and Yimer and Tadesse (2015). |
| 14 | Conceptual framework – pleases provide more detail in the text or the caption for Figure 1 on what is not depicted, e.g. variances for exogenous variables, etc. The conceptual framework can be more explicitly about why the authors expect women’s empowerment to be associated with child outcomes. How were the domains of women’s empowerment chosen? | Point taken. Correction has been made in the manuscript accordingly. |
| 15 | **Indicators:** Line 226, were only married women and women living with partner kept in the analysis? Several of the DHS indicators used in the measurement model are only collected for married women/women living with their partner. Indicate the cut-off used to define anemia. Women’s empowerment indicators – were “don’t know” responses coded as missing? The paragraph on attitudes towards wife beating was repeated, i.e. para lines 288-293, and para lines 294-300.  Please be specific throughout that household wealth quintile was included as a categorical variable. It is not always clear whether the continuous wealth index was used or the wealth quintile. | We considered both married women and women living with partner.  For anemia we, create a dummy variable indicating “0” as “ anemic” which include severe, moderate and mild as anemic level, and “1” if not anemic.  Yes, we considered “don’t know responses” as missing for each questionnaire of women’s empowerment indicators and replaced them with village average or mode responses. |
| 16 | Tables and figures - Please spell out all abbreviations in the main and supplemental tables and figures | The abbreviations have now been spelled out through put all figures and tables. |

# **References**

1.	Jones R, Haardörfer R, Ramakrishnan U, Yount KM, Miedema S, Girard AW. Women’s empowerment and child nutrition: The role of intrinsic agency. SSM-Population Health. 2019;9: 100475.

2.	Heckert J, Olney DK, Ruel MT. Is women’s empowerment a pathway to improving child nutrition outcomes in a nutrition-sensitive agriculture program?: Evidence from a randomized controlled trial in Burkina Faso. Social Science & Medicine. 2019;233: 93–102.

3.	Kuche D, Moss C, Eshetu S, Ayana G, Salasibew M, Dangour AD, et al. Factors associated with dietary diversity and length‐for‐age z‐score in rural Ethiopian children aged 6–23 months: A novel approach to the analysis of baseline data from the Sustainable Undernutrition Reduction in Ethiopia evaluation. Maternal & child nutrition. 2020;16: e12852.

4.	Na M, Jennings L, Talegawkar SA, Ahmed S. Association between women’s empowerment and infant and child feeding practices in sub-Saharan Africa: an analysis of Demographic and Health Surveys. Public health nutrition. 2015;18: 3155–3165.

5.	Yimer F, Tadesse F. Women’s empowerment in agriculture and dietary diversity in Ethiopia. International Food Policy Research Institute (IFPRI); 2015.

6.	Fantahun M, Berhane Y, Wall S, Byass P, Högberg U. Women’s involvement in household decision‐making and strengthening social capital—crucial factors for child survival in Ethiopia. Acta paediatrica. 2007;96: 582–589.

7.	Alemayehu YK, Theall K, Lemma W, Hajito KW, Tushune K. The role of empowerment in the association between a woman’s educational status and infant mortality in Ethiopia: Secondary analysis of demographic and health surveys. Ethiopian journal of health sciences. 2015;25: 353–362.

8.	Ebot JO. “ Girl Power!”: The Relationship between Women’s Autonomy and Children’s Immunization Coverage in Ethiopia. Journal of Health, Population and Nutrition. 2015;33: 18.

9.	Wado YD. Women’s autonomy and reproductive health-care-seeking behavior in Ethiopia. Women and Health. 2018;58: 729–743. doi:10.1080/03630242.2017.1353573

10.	Tiruneh FN, Chuang K-Y, Chuang Y-C. Women’s autonomy and maternal healthcare service utilization in Ethiopia. BMC health services research. 2017;17: 718.

11. StataCorp. 2015. Stata Statistical Software: Release 14. College Station, TX: StataCorp LP.

12. United Nations Children’s Fund (UNICEF), World Health Organization, International Bank for Reconstruction and Development/The World Bank. Levels and trends in child malnutrition: key findings of the 2019 Edition of the Joint Child Malnutrition Estimates. Geneva: World Health Organization; 2019 Licence: CC BY-NC-SA 3.0 IGO.

13. World Health Organization United Nations Children’s Fund (UNICEF), 2019. Levels and trends in child malnutrition: Key findings of the 2019 edition of the joint child malnutrition estimates.w§§1A

14. United Nations Children’s Fund (UNICEF), World Health Organization. “Levels and Trends in Child Malnutrition: Key Findings of the 2019 Edition of the Joint Child Malnutrition Estimates,” 2019.

15. Mabsout R. Capability and Health Functioning in Ethiopian Households. Social Indicators Research. 2011;101: 359–389. doi:10.1007/s11205-010-9661-0

16. Ndaimani A, Mhlanga M, Dube-Mawerewere V. The association between women’s empowerment and uptake of child health services: a demographic and health survey-based synthesis. Rockville, Maryland, USA: ICF; 2018. Available: http://dhsprogram.com/pubs/pdf/WP139/WP139.pdf
