# Supplementary material for: Associations between women’s empowerment and children’s health status in Ethiopia
Source: PLoS One. 2020 Jul 20;15(7):e0235825. doi: 10.1371/journal.pone.0235825 (PMC7371184; doi:10.1371/journal.pone.0235825)
Supplement: Attachment — Submitted filename: Response to Reviewers_2.docx [file pone.0235825.s006.docx]

**Response to Reviewers**

We appreciate the editorial response and the constructive and useful reviewer comments during both the first and second revision. We have incorporated comments and suggestions of the second round in the revised manuscript. The detailed responses to reviewer comments are provided below. For easy reference, we repeat the reviewer's comments in italics.

1. *EFA/CFA - conducting EFA and CFA on the full sample is a bit unorthodox. Typically, one random split half is used for EFA, and the remaining half for CFA. It would be helpful if the authors provide further rationale or a reference for using the full sample for these analyses.*

**Response:**

The reviewer is right in noting that the conventional approach for implementing CFA and EFA is using a split sample. However, we refer some studies and the studies indicated that implementing the EFA and CFA analyses using a full sample and split sample leads to similar results as far as methodological explanations can account for cases in which EFA and CFA lead to different conclusions based on the same sample (see e.g., (Van Prooijen & Van Der Kloot, 2001). These methodological issues include inadequate evidence on the indicators to measure the component, conservativeness of the CFA model and inappropriate applications of CFA. We address the methodological issues in measuring the indicators related to women’s empowerment as follows: first, based on previous literature we operationalized women’s empowerment variables to check whether the indicators related to women’s empowerment variable represent an existing entity that can be categorized in the same underlying latent factors. For example, women’s participation in household decision making have four indicators. Before considering these indicators in one latent dimension, first we coded the responses for each indicator and dichotomized them to know if the women is empowered or not (see section 4.1.2 of the manuscript). Second, some of the indicators related to women’s empowerment, such as women’s socio-economic status indicators, are not known in literature. Therefore, we performed EFA first. Then CFA is performed. (see section 4.21 line 476-486)

2. *Supplemental table 1 - I suggest you move this table to the main text. Rereading the paper now, I find myself wanting to see a description of the sample.*

**Response:** We have now added Table 1 to section 5 of the main manuscript. We also included an accompanying description of the table (see section 5.1).

3. *Introduction - thank you for including the additional literature. This is very helpful in putting your study in context. Lines 99-102, I agree that this is a limitation of the previous literature, but some the studies never aimed to assess infectious disease or causes of death, e.g. Heckert et al. aimed to asses only nutritional status. I suggest the authors re-phrase this sentence to more properly cite the literature. Lines 152-172, very helpful paragraph, thank you for including. It would be good to summarize the findings from these studies, rather than just reporting on what the studies examined. Since the para focuses on Ethiopia; I suggest you drop Heckert et al. which was conducted in Burkina.*

**Response:** We agree with the reviewer that Heckert et al., 2019 considered only child nutritional outcome. Hence, we have dropped the reference. We have also (i) re-phrased the sentence and (ii) summarized and wrote in one paragraph the main findings with a new paragraph.

*4. Methods - line 269, you only include married or co-habitating women, but then you control for marital status in you model. The variable in table 2 is called "married". What is the reference category? Co-habitating? Please clarify in the text.*

**Response:** In this study, we considered not only women who are currently married and living with partner but also other categories of marital status of such as widowed, divorced and separated. The variable marital status in our case is categorized as 1 if the women is married (stands for married or living with partner categories), 0 otherwise. In the DHS survey, women - either married or not married - responded to the questionnaire related to women’s empowerment variables and hence we use all women sample in our analysis. Brief description of this has been provided in the revised version of the manuscript (see section 4.1).

*5. Methods - lines 388-295, please define acceptable cut-offs for SRMR and CD, which you report.*

**Response:** Acceptable cut of value for SRMR is 0.08 or lower. The closer to 0, the better the fit of the model is (Hu & Bentler, 1999). A perfect correspond to Coefficient of Determination (CD) is 1. The closer the value of CD to 1, the better the goodness of fit of the model. Literatures indicated that the acceptable cut off value of CD is depending on the number of exogenous latent variables. Values of 0.67, 0.33 and 0.19 for endogenous latent variables in the inner path model are described as substantial, moderate and weak (Chin, 1998). (Henseler, Ringle, & Sinkovics, 2009) suggested that if endogenous latent variable explained by only a few exogenous latent variables, “Moderate” CD may be acceptable. If the endogenous latent variable relies on several exogenous latent variables, the CD value should exhibit at least a substantial level. We have now included this in text revised version of the manuscript (see section 4.2.1 line 542-550)

*6. Methods - in re-reading the paper I realized the authors have not addressed the issue of clustering. Typically, DHS analyses account for clustering at the EA level.*

**Response:** We have taken this reviewer’s comment and addressed the issue of clustering through clustering the standard errors at EA level. The result and the sign of the coefficient is not changed after considering clustering, but we presented the results with clustering (see Table 3).

*7. Methods - sample size - you report that you restrict analyses to 10,641 women (line 267) but table 2 has fewer observations. Were observations dropped due to missing data? please describe in the methods.*

**Response:** Our final sample size for the analysis depends on the children’s health outcome variables. So, the lower observation in some of the models is due to missing values (observations) for the outcome variable under consideration. We have not explained this point in the manuscript (as a note to Table 3) to make it clear to the reader.

8. Discussion - several times the authors say empowerment is important for "child development", but child development was not a measure included in the paper.

**Response:** Point taken. We replaced the word “child development” with appropriate outcome variables throughout the paper.

**References**

Chin, W. W. (1998). The partial least squares approach to structural equation modeling. *Modern Methods for Business Research*, *295*(2), 295–336.

Henseler, J., Ringle, C. M., & Sinkovics, R. R. (2009). The use of partial least squares path modeling in international marketing. In *New challenges to international marketing*. Emerald Group Publishing Limited.

Hu, L., & Bentler, P. M. (1999). Cutoff criteria for fit indexes in covariance structure analysis: Conventional criteria versus new alternatives. *Structural Equation Modeling: A Multidisciplinary Journal*, *6*(1), 1–55.

Van Prooijen, J.-W., & Van Der Kloot, W. A. (2001). Confirmatory analysis of exploratively obtained factor structures. *Educational and Psychological Measurement*, *61*(5), 777–792.
